# A Rare Anatomical Variant Dual Radial Digital Nerves in the Right Ring Finger: Consideration in Hand Dissection

**DOI:** 10.1016/j.jhsg.2025.100906

**Published:** 2026-01-09

**Authors:** Nikolas Vlassis, Janse T. Schermerhorn, Genevieve Rambau

**Affiliations:** ∗ Uniformed Services University of the Health Sciences, F. Edward Hébert School of Medicine, Bethesda, MD; † Department of Orthopedics, Walter Reed National Military Medical Center, Bethesda, MD

**Keywords:** Anatomical variants, Digital nerves, Dupuytren, Hand anatomy, Neurovascular bundle

## Abstract

Digital nerve anatomy in the fingers is considered a predictable pattern of a single radial and ulnar neurovascular bundle, allowing for often safe midline dissection. In complicated cases, such as Dupuytren contracture or trauma, knowledge of digital nerve anatomy is paramount for safe dissection. The patient was a 68-year-old man with bilateral ring finger Dupuytren contractures. On the right hand, he had a 40° flexion contracture of the ring finger and underwent a palmar fasciectomy. Two distinct radial digital nerves were identified intraoperatively, with one branch in the traditional radial aspect of the digit, and a second branch centrally overlying the flexor tendons. This finding was later corroborated on the contralateral ring finger when he underwent subsequent palmar fasciectomy. Dual radial digital nerves are an uncommon anatomical variant that underscores the importance of careful neurovascular dissection. This case also raises concerns about percutaneous procedures distal to the metacarpophalangeal joint crease.

Digital nerve anatomy is often considered straight forward in the digit with a distinct ulnar and radial neurovascular bundle. Its predictable pattern allows for swift and safe midline dissection in both normal anatomy and abnormal anatomy (eg, trauma, prior surgery, palmar fibrosis). It is well known of the risks with aberrant anatomy in cases of Dupuytren contracture, where the nerves may be displaced volarly and centrally attributed to a spiral cord and in these instances, localization of the digital nerves is paramount to avoiding injury. The neurovascular bundles often act as a “beacon” for safety, and once identified and protected, allowing for swift excision of the diseased fascia. Addition nerve variations are extremely rare and unexpected, and unless there is diligence in careful dissection, can easily result in injury.

This case report discusses a unique finding of dual radial digital nerves in the right ring finger during a routine palmar fasciectomy, emphasizing the importance of recognizing and managing such variants to ensure surgical success and patient safety.

## Case Report

A 68-year-old right-handed man presented with a progressive 40° flexion contracture of the right ring finger at the metacarpophalangeal joint. The patient reported difficulty performing activities of daily living, including placing his hand in his pocket and grasping objects. Conservative treatments, including collagenase clostridium hisolyticum injections, had failed to provide lasting improvement. After thoroughly discussing the risks, benefits, and alternatives, the patient proceeded with surgical intervention.

**Figure 1:**
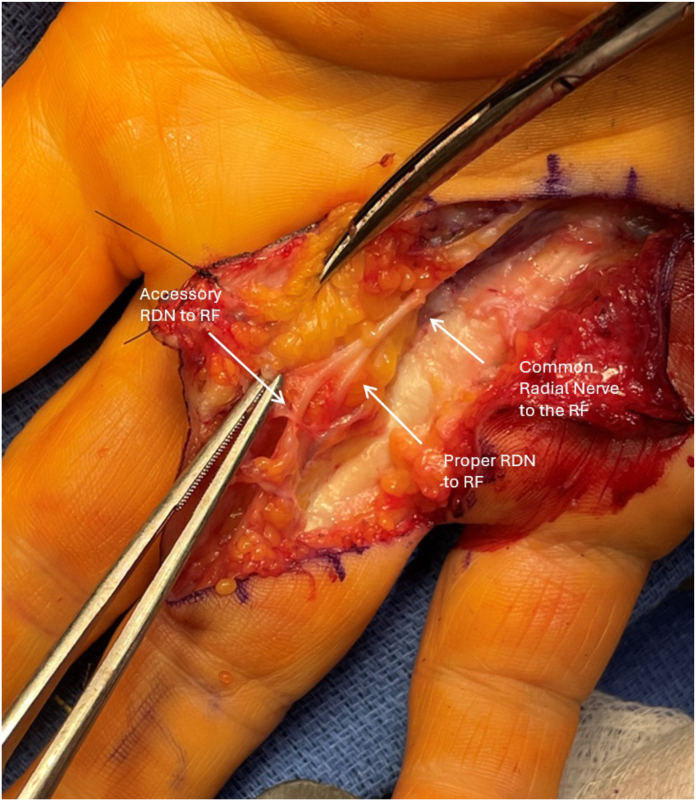
Gross clinical intraoperative picture of the dual radial digital nerves of the right long finger reflected back. RDN, radial digital nerve; RF, ring finger.

**Figure 2:**
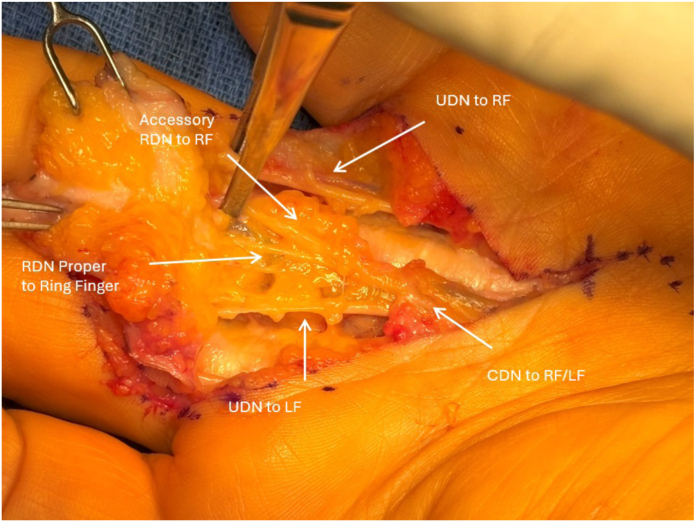
Image showing the common digital nerve to the ring and long finger with a second dual radial digital nerve. CDN, common digital nerve; LF, long finger; RDN, radial digital nerve; RF, ring finger; UDN, ulnar digital nerve.

A Brunner-style incision was made from the proximal palmar crease overlying the A1 pulley to the proximal interphalangeal joint crease. The subcutaneous tissues were meticulously dissected, exposing the thickened palmar fascia. Localization of the radial and ulnar neurovascular bundles was then performed to allow for safe protection during cord excision. During dissection, a digital nerve was encountered midline overlying the flexor tendons. As the ulnar digital nerve had already been isolated, it was assumed to the be the radial digital nerve (Fig. 1). This was carefully traced back to the metacarpophalangeal (MCP) joint crease where it was found to join the radial nerve proper. The radial digital nerve proper was then isolated in its traditional location on the radial border of the digit. After carefully separating off the diseased fascia, the anatomy clearly showed a common radial digital nerve dividing into two distinct nerves with one following the classic pathway and another centrally (Fig. 2). The ulnar digital nerve had normal anatomy and location. The cord was identified as a spiral cord, consistent with the observed volar and central displacement of the neurovascular structures. The palmar cord and associated nodule were excised. After releasing the tourniquet, the digit was well-perfused and demonstrated full extension with approximately 45° of hyperextension at the metacarpophalangeal joint. The incision was closed, and the hand was immobilized. After surgery, the patient was discharged without complications. The patient recovered well after surgery and elected to proceed with cord excision of the ring finger on the left hand. The case proceeded in a similar fashion as described above, and the patient was found to have the same anatomy on the left hand with two radial digital nerves and a single ulnar digital nerve. He recovered uneventfully with a normal table top examination at two months of follow-up. He has normal sensation in both digits with no neurovascular deficits. The patient consented to dissemination of his case description.

## Discussion

Variations in neurovascular anatomy are uncommon but well-documented in hand surgery.^1^ More distally, variations in digital nerve branching are common. However, based on the author’s extensive literature review, the presence of two distinct radial digital nerves, despite the presence of a distinct ulnar digital nerve in this patient’s right ring finger, has never been described.^2^ Based on the author’s extensive literature review, this accessory nerve variant has never been described. Aberrant anatomy like this can have critical surgical implications. We present this case to emphasize the importance of maintaining vigilance for this particular anatomical variant, as well as others. In this case, failure to recognize the accessory radial digital nerve could have resulted in iatrogenic injury and subsequent sensory deficits or chronic pain (Figs. 1 and 2). The successful outcome highlights the importance of intraoperative diligence and attention to detail.

It is unclear if one or both of these nerves provide sensory innervation to the digit and, further, what their individual contributions are proportionally, if so. However, this finding prompts further concern about percutaneous procedures particularly in the setting of Dupuytren contracture. Percutaneous needle aponeurotomy and collagenase clostridium hisolyticum injections have their own indications and role in the treatment of Dupuytren cords. These procedures are often recommended to be limited to within 2–3 mm distal of the MCP joint crease. However, in this patient, the accessory radial digital nerve was midline at the level of the MCP joint crease and could easily have been injured in one of these procedures. Although these variants are rare, this underscores the importance of counseling patients on the risks of these procedures and may alter indications and limitations to each. Although it is unclear what deficits may be incurred with injury to the accessory nerve, sequelae of neuroma or other chronic pain could result. This finding may help avoid these unnecessary outcomes.

The obvious limitation of this case report is that it is a single case describing this anatomical variant in one patient, although it was confirmed bilaterally. We suggest that other case reports or series should be published to understand better the incidence of duplicate digital nerves across the population.

This case report highlights a rare anatomical variant of an accessory radial digital nerve encountered during Dupuytren contracture surgery. Identifying and carefully managing this anatomic variant ensured a successful surgical outcome without neurologic compromise. Surgeons should remain vigilant for such variations to optimize patient safety and surgical efficacy. Further case series are needed to better understand this anatomical variant’s prevalence in the population.

## Disclaimers

The opinions and assertions expressed herein are those of the author(s) and do not reflect the official policy or position of the Uniformed Services University, Walter Reed National Military Medical Center, the Departments of the Navy or Air Force, the Department of Defense, or the US Government.

## Conflicts of Interest

No benefits in any form have been received or will be received related directly to this article.
